# Examining 12 Years of Pedestrian Injuries and Fatalities in Wichita, Kansas: A Retrospective Study

**DOI:** 10.7759/cureus.80766

**Published:** 2025-03-18

**Authors:** Amanda I Aguila Gonzalez, Elizabeth Ablah

**Affiliations:** 1 Department of Population Health, University of Kansas School of Medicine - Wichita, Wichita, USA

**Keywords:** motor vehicle crash (mvc), pedestrian, pedestrian vs auto, safety, severe injury, traffic fatality

## Abstract

Background

As more people walk and bike in their communities, there is a corresponding increase in the number of crashes with motor vehicles. Each year, a significant number of pedestrians die in motor vehicle crashes in the U.S., and many more are injured. Safety interventions to decrease motor vehicle crashes have included roadway design, safety programs, and laws. However, there is still much that is unknown about the factors that impact motor vehicle crashes with pedestrians. This study aimed to describe the pedestrian motor vehicle crashes occurring in Wichita, Kansas, from 2008 through 2019, specifically to identify factors associated with fatalities/serious injuries of pedestrians.

Methods

This study was a retrospective analysis of data from the Kansas Motor Vehicle Accident Reports from January 2008 through December 2019. Bivariate and multivariate logistical regression analyses were conducted to determine factors associated with fatalities/serious injuries of pedestrians from motor vehicle crashes.

Results

From 2008 through 2019, 979 motor vehicle crashes involving a pedestrian were reported, with an upward trend of crashes reported over the years. Of the total motor vehicle crashes reported, 97.5% (n = 955) resulted in a pedestrian injury. In 67.3% (n = 659) of motor vehicle crash cases involving a pedestrian, medical assistance and transport to the nearest hospital occurred. The severity of injury varied per crash; 18.2% (n = 177) of crashes resulted in a severe or fatal injury for a pedestrian. There were significant associations between pedestrian serious/fatal injuries and pedestrian age, driver age, time of day, road characteristics, vehicle maneuver, vehicle damage, pedestrian location before impact, pedestrian substance abuse, and driver substance abuse.

Conclusions

The frequency of pedestrian crashes in Wichita either remained consistent or increased over the course of 12 years, underscoring a need for action to increase safety and implement policy efforts to decrease crash prevalence. Further exploration of pedestrian crashes through a multilevel model can yield knowledge of individual behaviors and environmental factors contributing to crashes.

## Introduction

Motor vehicle crashes involving pedestrians are a public health concern worldwide, as there is an increase in the number of pedestrian crashes each year [1,2]. More than 5,000 pedestrians die in a motor vehicle crash each year in the U.S. [3], and more than 50,000 are injured [4].

A study by Buehler and Pucher [3] assessed changes in pedestrian fatality rates per 100,000 population from 1990 through 2014 for 11 countries, including the U.S. The findings indicate that pedestrian fatality rates decreased across all countries. However, the U.S. showed the least progress, with a 35% decrease in pedestrian fatalities compared to Canada (49%), Japan (52%), Australia (75%), and other Western European countries [3]. Another study indicates that there was a slow increase in fatalities across the U.S. from 2009 through 2018 [5]. Between 2008 and 2017, national pedestrian fatalities increased by 32% [6], including 6,283 pedestrians killed in crashes with motor vehicles [6]. Pedestrian injuries from collisions with motor vehicles increased by 7.7%, and pedestrian fatalities increased by 9.5% between 2014 and 2015 alone [7]. This increase in deaths is the second greatest among all road users (e.g., drivers and motorcyclists) [8].

In 2016, 6,080 pedestrians were killed in a motor vehicle crash [9]. By 2017, the number of deaths slightly decreased to 5,977 pedestrian deaths, which accounts for 16.1% of the 37,133 people killed in motor vehicle crashes in the U.S. [9]. In 2019, the U.S. average pedestrian fatality rate was 1.91 per 100,000 population [5]. In 2020, this rate increased slightly to 1.99 per 100,000 reported fatalities [10].

Pedestrian fatalities by state

In Kansas, the pedestrian fatality rate was 1.17 per 100,000 population in 2017 [11]. By 2018, the Kansas pedestrian fatality rate had decreased to 0.96 per 100,000 population [5]. In 2019, the Kansas pedestrian fatality rate continued to decrease to 0.55 per 100,000 population [12]. However, by 2020, the Kansas pedestrian fatality rate more than doubled to 1.60 per 100,000 population [10].

Pedestrian prevention in Wichita, Kansas

Pedestrian crash prevention efforts in Kansas have focused primarily on education and allocating funds for small preventive efforts in some cities [12]. The prevention effort has been passive, including the distribution of brochures and the creation of radio advertising [5]. Wichita is the largest city in Kansas; as of 2020, Wichita’s population was 397,532 [13]. Additionally, 81,932 residents live outside of Wichita but commute to work in the city, and 56,978 live in Wichita but work outside the city [13]. Therefore, safety and prevention efforts to mitigate motor vehicle crashes with pedestrians are imperative. Although it is critical to understand the characteristics of motor vehicle crashes with pedestrians, little is known about the characteristics associated with these types of crashes. The objective of this study was to determine what factors were associated with pedestrian motor vehicle crashes that result in serious or fatal injuries.

## Materials and methods

Study design

This study followed a retrospective study design because the objective was to determine what factors were associated with pedestrian motor vehicle crashes that resulted in serious or fatal injuries in the city of Wichita, Kansas, from 2008 through 2019. Data for this study were already collected as part of police accident reports. These reports were completed as part of standard police procedures when a motor vehicle crash was reported. A retrospective study design allowed for timely and cost-effective analysis. Through a partnership with the Wichita Police Department, all crash reports were converted into digital PDF files for analysis. Crash reports with missing information, file errors, or that did not involve a pedestrian were excluded from this study. The inclusion criteria were motor vehicle crashes involving a pedestrian.

Data

Data from this study included all pedestrians and drivers documented in a crash in the Kansas Motor Vehicle Accident Reports collected by the Wichita Police Department from January 2008 through December 2019. All data were collected in person by police officers when a crash occurred. A “crash” was defined as an unstable situation involving a vehicle in transport [14]. A “crash” can also be referred to as a “motor vehicle crash” and defined as an incident that involves one or more motor vehicles, where at least one vehicle was in transport and the crash originated on a public trafficway, such as a road or highway [15]. For this study, a motor vehicle crash was defined as involving one motor vehicle and a pedestrian. Motor vehicles included in this study were cars, pickups, sport utility vehicles (SUVs), trucks, and buses. Vehicles in transport were defined primarily as vehicles in motion on a roadway or in motion outside a trafficway.

Instrument

All records pertaining to the Wichita Police Department’s Kansas Motor Vehicle Accident Report from 2008 through 2019 were recorded by case number. All reports followed a standard police crash report format, including information about the crash date, pedestrian and/or driver demographic information (e.g., date of birth, age, and gender), whether there was an injury or death, and other details about the crash. Criteria for inclusion were motor-vehicle crashes that involved a pedestrian. According to the Kansas Law Enforcement Crash Report Coding Manual [14], pedestrians were defined as persons who are not occupants of motor vehicles in transport. In this context, a pedestrian was defined as any person on foot, walking, running, jogging, hiking, sitting, or lying down who was involved in a motor vehicle crash [15].

Injury and fatality

Data abstracted regarding the severity of the injury for each occupant (including driver, passenger, and/or pedestrian) were defined according to the Wichita Police Department Law Enforcement Crash Report Coding Manual [14]. These strata included: (1) No apparent injury - no physical evidence of an injury or the involved parties (i.e., pedestrian, driver, and passenger) do not report any. (2) Possible injury - any injury reported or claimed which is not fatal, suspected as minor or serious (e.g., limping, complaint of pain, and nausea). (3) Minor injury or non-incapacitating injury - injury evident at the scene of the crash other than fatal or serious injuries (e.g., abrasions, bruises, and minor lacerations). (4) Suspected serious injury or disabling - any injury other than fatal, which results in a severe laceration, broken or distorted extremity, significant burns, unconsciousness, or paralysis.

Any person(s) killed in or outside of any vehicle involved in the crash, or who died within 30 days of a crash as a result of an injury sustained from the crash, was considered a fatality [14]. Injury severity was recoded from a categorical variable with five levels (no apparent injury, possible injury, minor injury or non-incapacitating injury, suspected serious injury, fatal injury) to a dichotomous variable, labeled “severe or fatal injury,” with “yes” and “no” as potential responses. This recoding allowed for the use of logistic regression analysis to identify variables associated with pedestrian motor vehicle crashes.

Medical assistance data were also abstracted; these data included whether a pedestrian was treated on the scene of the crash, required a doctor’s visit, or needed immediate medical assistance, such as admission to the hospital via emergency vehicle. Finally, data were abstracted regarding fatalities. If there was a death, details were abstracted, such as whether the death occurred immediately upon crash or during or after transport. Additionally, data abstracted included whether a hit and run occurred, citations, and licensure status.

Procedures and analysis

This study was approved by the Human Subjects Committee at the University of Kansas School of Medicine - Wichita. A database was created utilizing IBM SPSS Statistics for Windows, Version 26 (Released 2019; IBM Corp., Armonk, NY, USA), in which all crash incident reports from 2008 through 2019 were abstracted.

To assess the relationships between pedestrian injuries or fatal crashes and various independent categorical variables, analyses were performed using Chi-square statistics. Independent variables included crash conditions, location, vehicle features, pedestrian behaviors and actions, citation, impairment, and crash fault. The severity of injury was recoded to be a dichotomous variable (i.e., severe injury - yes/no; fatality - yes/no). To determine which independent variables were associated with pedestrian serious or fatal injuries, a logistic regression was conducted.

## Results

**Figure 1:**
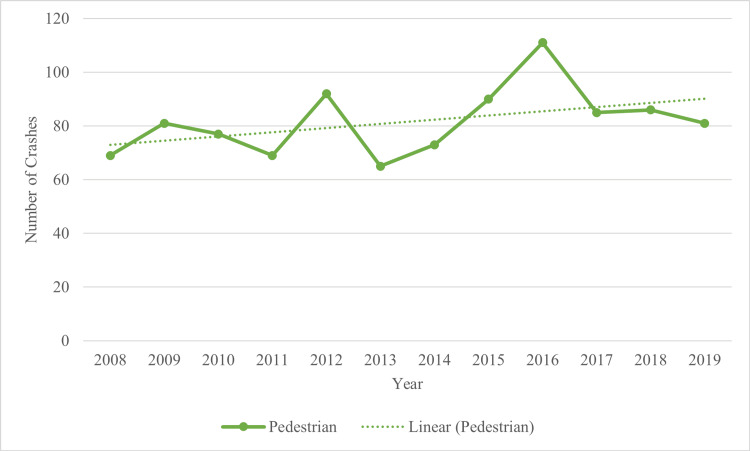
Crashes With Pedestrians in Wichita From 2008 Through 2019

A total of 2,036 motor vehicle crashes were reported from 2008 through 2019; crashes were excluded from this study due to missing information/file errors (n = 21) or crashes not involving a pedestrian (n = 1,036). The final sample size was 979 motor vehicle crashes involving a pedestrian. The data generally illustrated an upward trend of crashes reported over the years (Figure 1), suggesting an overall increase in crashes involving pedestrians in Wichita, Kansas, over 12 years. The lowest number of reported crashes for pedestrians was in 2013 (n = 65). An increase in pedestrian crashes occurred in 2014 (n = 73), reaching its highest point in 2016 (n = 111), with the highest count of pedestrian crashes reported in 12 years.

Demographics

Nearly half of pedestrian crashes involved a male driver (46.4%, or n = 454; Table 1). Additionally, in 63.7% (n = 624) of pedestrian crashes, the pedestrian involved was also male. Less than 1% of the drivers in pedestrian crashes reported being younger than the legal driving age for Kansas (i.e., 14 years). Further, 6.7% (n = 66) of drivers involved in pedestrian crashes reported being older than 70 years.

**Table 1:** Pedestrian and Driver Demographics

	Pedestrian Crashes, % (n)
Pedestrian, gender	
Male	63.7 (624)
Female	36.0 (352)
Missing	0.3 (3)
Total	100.0 (979)
Age (in years)	
1 to 10	12.2 (119)
11 to 20	20.0 (195)
21 to 30	17.4 (170)
31 to 40	12.7 (124)
41 to 50	13.6 (133)
51 to 60	11.1 (109)
61 to 70	8.3 (81)
71+	3.4 (33)
Missing	1.3 (15)
Total	100.0 (979)
Driver, gender	
Male	46.4 (454)
Female	39.7 (389)
Missing	13.9 (136)
Total	100.0 (979)
Age	
14 or younger	0.06 (4)
15 to 25	15.1 (148)
26 to 36	15.3 (150)
37 to 47	13.9 (132)
48 to 58	12.0 (117)
59 to 69	9.5 (93)
70+	6.7 (66)
Missing	27.44 (269)
Total	100.0 (979)

Hit-and-run crashes

**Figure 2:**
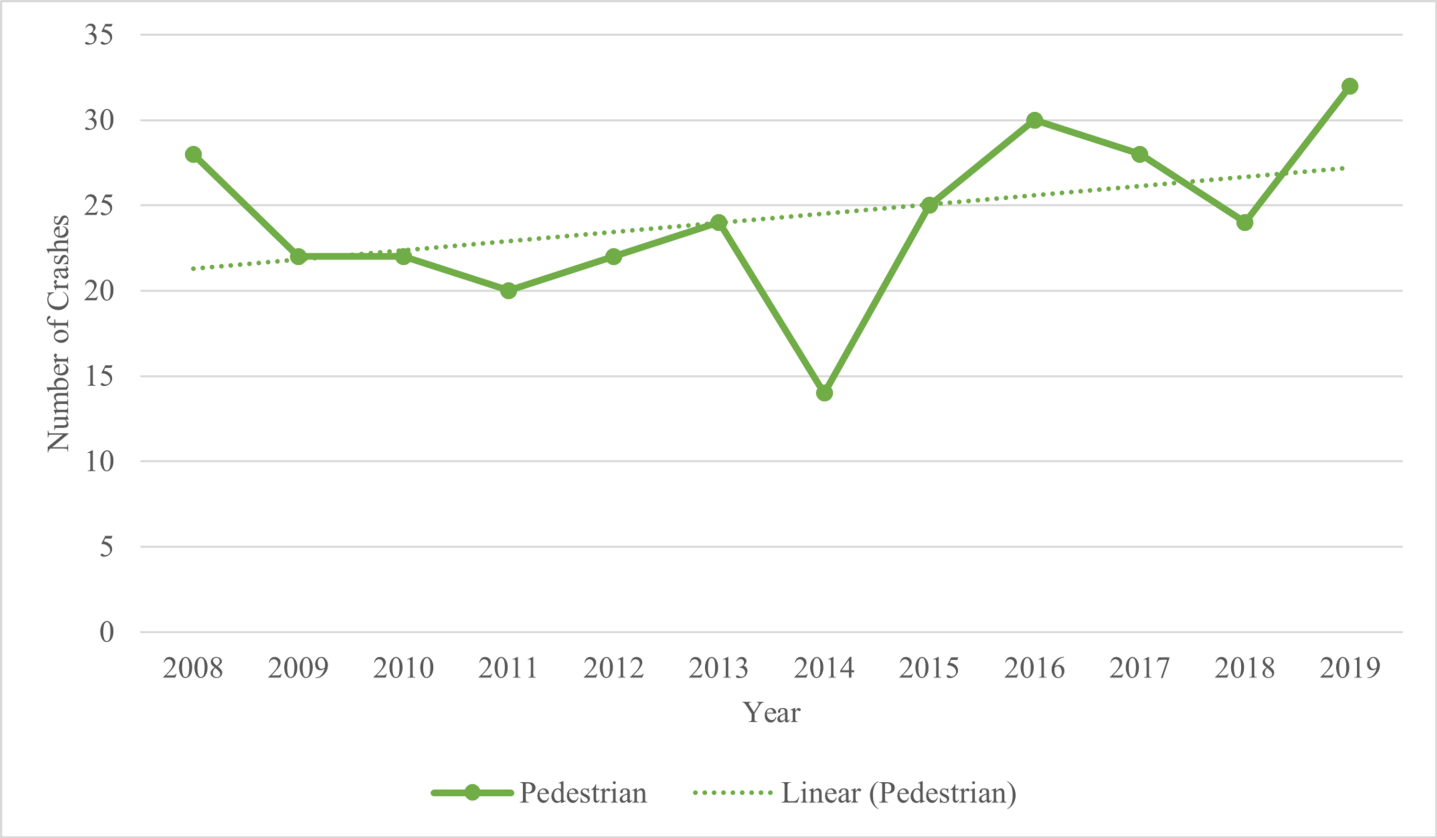
Hit-and-Run Crashes by Year

It is important to note that 28.1% (n = 291) of crashes involving a pedestrian were reported as hit-and-run crashes (Figure 2). Between 2008 and 2013, the prevalence of hit-and-run crashes involving pedestrians was relatively steady, with an average of 23 (SD = 2.517) crashes each year, reaching the lowest frequency in 2014 (n = 14). However, from 2014 through 2016, the number of pedestrian hit-and-run crashes more than doubled (n = 32).

Injuries and fatalities

Of the total number of pedestrian crashes reported, 97.5% (n = 955) resulted in a pedestrian injury. The severity of injuries varied by crash (Table 2).

**Table 2:** Pedestrian and Driver Injuries and Fatalities

	Pedestrian Crashes, % (n)
Pedestrian	
Possible injury	31.5 (305)
Suspect minor injury	48.8 (473)
Suspect serious injury	13.9 (135)
Fatal injury	4.3 (42)
No apparent injury	1.4 (14)
Total	100.0 (969)
Driver	
Possible injury	0.7 (5)
Suspect minor injury	1.3 (10)
Suspect serious injury	0.1 (1)
Fatal injury	0.0 (0)
No apparent injury	97.9 (740)
Total	100.0 (756)

**Figure 3:**
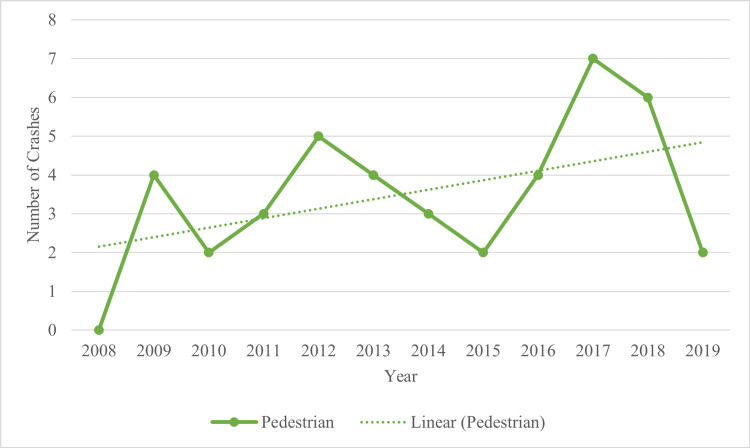
Pedestrian Fatalities From 2008 Through 2019

Of all motor vehicle crashes involving a pedestrian, 18.2% (n = 177) resulted in a pedestrian serious or fatal injury (Table 2). The median number of pedestrian fatalities from 2008 through 2019 was three, and the mode for pedestrian crash fatalities was four (Figure 3). In 67.3% (n = 659) of motor vehicle crashes involving a pedestrian, medical assistance and transport to the nearest hospital occurred.

The objective of this study was to determine what factors were associated with pedestrian serious/fatal injuries when involved in a motor vehicle crash. Pearson Chi-square analyses were conducted to determine if there were significant associations between motor vehicle crashes that resulted in a pedestrian serious or fatal injury and selected independent variables. Significant associations were found between pedestrian serious/fatal injury and pedestrian age, driver age, time of day, road characteristics, vehicle maneuver, vehicle damage, pedestrian location before impact, pedestrian substance use, and driver substance use (Table 3).

**Table 3:** Pedestrian Serious or Fatal Injuries Bivariate Analyses

	% (n)	p-value
Pedestrian age		0.002
Child (0 to 14 years)	15.8 (28)	
Youth (15 to 24 years)	15.8 (28)	
Adult (25 to 64 years)	54.2 (96)	
Older adult (65 years or older)	14.1 (25)	
Total	100.0 (177)	
Driver age		0.014
Child (0-14 years of age)	0.7 (1)	
Youth (15-24 years of age)	26.5 (39)	
Adult (25-64 years of age)	54.4 (80)	
Older adult (65 years of age or older)	18.4 (27)	
Total	100.0 (147)	
Time of day		<0.001
Daylight	44.9 (79)	
Dark	55.1 (97)	
Total	100.0 (176)	
Road characteristics		0.024
Not straight and leveled	4.5 (8)	
Straight and leveled	95.5 (168)	
Total	100.0 (176)	
Vehicle maneuver		0.004
Straight	76.1 (124)	
Turning or passing	23.9 (39)	
Total	100.0 (163)	
Vehicle damage		<0.001
Yes	85.3 (128)	
No	14.7 (22)	
Total	100.0 (150)	
Pedestrian location before impact		<0.001
Not in crosswalk	76.9 (133)	
In crosswalk	23.1 (40)	
Total	100.0 (173)	
Pedestrian substance use		<0.001
Yes	10.9 (17)	
No	89.1 (139)	
Total	100.0 (156)	
Driver substance use		<0.001
Yes	5.6 (9)	
No	94.4 (151)	
Total	100.0 (160)	

A logistic regression was performed to ascertain the association of pedestrian age, driver age, time of day, road characteristics, vehicle maneuver, vehicle damage, pedestrian location before impact, and pedestrian and driver substance use on the likelihood that a pedestrian experienced a serious or fatal injury from a crash with a motor vehicle. The logistic regression model was not statistically significant, and none of the independent variables significantly loaded into the regression model. The model explained 17.4% (Nagelkerke R²) of the variance in pedestrian serious or fatal injuries.

## Discussion

The objective of this study was to determine what factors were associated with pedestrian serious/fatal injuries when involved in a motor vehicle crash. Results indicate that the number of pedestrian crashes in Wichita either remained consistent or increased over 12 years. This research found a small number of specific streets and intersections that have had a high number of pedestrian injuries and fatalities, which provides an opportunity to reduce these numbers by implementing environmental and structural changes. Not only do these findings allow for changes aimed at reducing injuries and fatalities, but they also allow the opportunity to increase awareness regarding safety and prevention measures for pedestrians. Variables associated with motor vehicle crashes at the bivariate level that resulted in a serious or fatal pedestrian injury included pedestrian age, driver age, time of day, road characteristics, vehicle maneuver, vehicle damage, pedestrian location before impact, pedestrian substance use, and driver substance use. This study suggests that adult drivers and pedestrians between the ages of 25 and 64 years were more likely to be involved in a crash that resulted in a serious or fatal injury between 2008 and 2019 than children or youth. Additionally, most serious or fatal pedestrian injuries from a motor vehicle crash occurred when conditions were dark, suggesting that visibility may have played a role in pedestrian crashes that resulted in a serious or fatal injury. The majority of these crashes occurred when roadways were straight and leveled. Further, in most pedestrian serious or fatal crashes, the driver was driving straight at the time of the crash, and their vehicle was still functional, with minimal to no damage. In motor vehicle crashes in which a pedestrian sustained a serious or fatal injury, the pedestrian was not in a crosswalk at the time of the crash, but rather in an area or intersection without a crosswalk. This finding is consistent with national trends on pedestrian crashes [16]. Additionally, pedestrian serious or fatal injuries that occurred from 2008 through 2019 were not the result of pedestrian or driver impairment, as most of the crash reports indicated no evidence of impairment from either party.

Most motor vehicle crashes with pedestrians resulted in injuries, and this study suggests that medical assistance was required in most cases, further echoing the need for additional safety measures to prevent crashes. Pedestrian fatalities resulting from motor vehicle crashes decreased from 2012 through 2015. However, from 2015 through 2017, pedestrian fatalities more than doubled, reaching the highest point in 12 years in 2017. Further retrospective studies assessing factors associated with a decrease in crashes from 2012 through 2015, and how these differ from crashes that occurred between 2015 and 2017, may yield a better understanding of environmental and infrastructure-related condition changes that created the spike in crashes.

This study is consistent with national pedestrian traffic safety data that illustrate a trend of increasing pedestrian fatalities, especially at night [17]. Additionally, most serious or fatal pedestrian injuries from a motor vehicle crash occurred when conditions were dark, suggesting that visibility may have played a role; this finding is consistent with the literature [17].

Despite the associations at the bivariate level, these relationships yielded no significant associations in a multivariable model. Regression analysis explained less than a quarter of the variance, suggesting that future research is needed to better understand the factors associated with pedestrian serious or fatal injuries resulting from a motor vehicle crash. The exploration of pedestrian crashes through a multilevel model can yield knowledge of individual behaviors and environmental factors that may have contributed to these crashes. To improve the safety of pedestrians, it is important to gain an understanding of crash-contributing factors, including the built environment, safety and prevention policies, characteristics of drivers, pedestrians, and crash events [18]. Further, there is a lack of continuity between systems, as police and health system reports may include different information that is relevant to understanding and preventing crash occurrence. This study's findings suggest that, in more than half of crashes, medical assistance was required. However, emergency medical assistance responders and hospitals have additional relevant information, such as whether the pedestrian required hospitalization, length of stay, or mortality, weeks or even months after the crash as a result of the injuries sustained in the crash. To improve road safety and prevent an increase in motor vehicle crashes with pedestrians, linkages among these data sources are critical.

The findings from this study suggest a need to focus on priority areas where a high number of crashes occurred, as well as on areas where crashes resulting in pedestrian serious or fatal injury occurred. Additionally, policy, prevention, and safety efforts, such as the adoption of policies like “stop-as-yield” [19] and/or “complete streets” [20], are needed to create safe spaces for community members to be active and reduce the number of crashes. Another policy worth consideration is a more rigorous licensing renewal process for drivers. A more rigorous licensing renewal process focused on cognitive skills testing for drivers may play a role in road safety for all road users and prevent future crashes. These results can help inform policies and plans to increase safety and make commuting by walking easier, safer, and more convenient. These efforts have already begun, as the findings from this study have been presented to city and regional planners, police department officials, pedestrian advisory groups, and non-profit organizations focused on prevention and advocacy for pedestrians. The findings have allowed these groups to shift their efforts to address safety in high-crash areas. It is important for roadway design engineers and other policy and decision-makers to consider the findings from this study, especially those that relate to high-crash areas and severe and fatal injuries, in their planning efforts. Street designs promoting safety for pedestrians and cyclists include designated spaces for these groups, such as sidewalk zones, pedestrian zones, buffer zones, medians, crosswalks, and other design elements focused on increasing safety.

Limitations

This study is subject to some limitations. First, results include crashes that were reported to the Wichita Police Department (i.e., crashes for which a report was filed). There were likely other crashes that were not reported, increasing the incidence of crashes highlighted in this study. Another limitation is that all data were abstracted from police accident report forms over 12 years, and during that time, the format of the police reports changed. Accordingly, officers had to adjust to new processes and coding for information to be able to complete the form, which may have contributed to some inaccuracies and missing data. It is also important to consider that the data may be susceptible to response bias and interview bias, as the officers completed forms with the pedestrians and/or drivers, conducting the interaction from a position of authority [21].

The lack of reporting of race and ethnicity data was noted as a study limitation, as these were not captured in the police accident report forms. Therefore, not knowing the racial and ethnic identities of pedestrians, drivers, and passengers, we cannot conclude if serious or fatal injuries may be associated with race or ethnicity. Additionally, historic racial trauma associated with police presence may have impacted the likelihood of the parties completing a police crash report and/or providing accurate accounts of crashes. A study by Lewis and Bueno de Mesquita [21], assessing racial differences in hospitalization (not specific to motor vehicle crashes) in Indianapolis, Indiana, and Wichita, Kansas, suggests that the presence of more than one police officer significantly reduced the odds of hospitalization after injury or serious injury. Therefore, depending on the factors at the time of the crash, the presence of multiple police officers could have resulted in a form of response bias.

Future research

This study focused on understanding factors associated with pedestrian crashes with motor vehicles that resulted in a serious or fatal injury. Future research needs to consider the role of vehicle speed in pedestrian serious and fatal injuries resulting from a crash. Assessing speed limits in high-crash areas and whether the driver was driving within that speed limit may yield valuable information to inform driving laws. Since determining vehicle speed can sometimes be a challenge when a crash has taken place, transportation engineers, planners, and other decision-makers in this space must consider the findings of this study regarding high-crash areas and determine appropriate speed limits for those areas. Further, it may be beneficial for stakeholders, such as transportation engineers and planners, to adapt walk audits or similar strategies and conduct “speed audits” in high-crash areas. This can be achieved by partnering with local organizations, such as non-profits, city officials, police departments, local businesses, or other groups who have a stake in transportation planning and pedestrian safety. A speed audit, both in real time and looking back at speeding citations, to determine changes in speed limits, driving laws, or enforcement, needs to occur. Additionally, future research must consider if there are opportunities for supplemental data collection outside of police report data to address potential response and social desirability biases that may occur with the presence of law enforcement.

## Conclusions

This study suggests an overall increase in pedestrian crashes in Wichita, Kansas, over 12 years. Nearly all crashes involving a pedestrian resulted in the pedestrian’s injury. Of the total motor vehicle crashes with available injury information, 18.2% (n = 177) resulted in a pedestrian’s serious or fatal injury. Variables associated at the bivariate level with motor vehicle crashes that resulted in a pedestrian’s serious or fatal injury included: pedestrian age, driver age, time of day, road characteristics, vehicle maneuver, vehicle damage, pedestrian location before impact, pedestrian substance use, and driver substance use. This study suggests that adult drivers and pedestrians between the ages of 25 and 64 years were more likely to be involved in a crash that resulted in a serious or fatal injury between 2008 and 2019 than children or youth. Additionally, most serious or fatal pedestrian injuries from a motor vehicle crash occurred when conditions were dark, suggesting that visibility may have played a role in pedestrian crashes that resulted in a serious or fatal injury. Further, the majority of these crashes occurred when roadways were straight and leveled. However, the pedestrian was not in a crosswalk at the time of the crash but rather in an area or intersection without a crosswalk. In addition, in most pedestrian serious or fatal crashes, the driver was driving straight at the time of the crash, their vehicle was still functional, with minimal to no damage, and there was no evidence of impairment from either party involved. Despite the associations at the bivariate level, these relationships yielded no significant associations in a multivariate model.

The exploration of pedestrian crashes through a multilevel model can yield knowledge of individual behaviors and environmental factors that may have contributed to these crashes. Exploring hospital data, along with police department data, would also allow insight into areas for pedestrian safety improvements and potentially mitigate biases noted in limitations. To improve safety, it is important to gain an understanding of crash-contributing factors, including the built environment, safety and prevention policies, characteristics of drivers, pedestrians, and crash events.
